# Determinants of malaria infections among children in refugee settlements in Uganda during 2018–2019

**DOI:** 10.1186/s40249-023-01090-3

**Published:** 2023-04-10

**Authors:** Henry Musoke Semakula, Song Liang, Paul Isolo Mukwaya, Frank Mugagga, Monica Swahn, Denis Nseka, Hannington Wasswa, Patrick Kayima

**Affiliations:** 1 Department of Geography, Geo-informatics and Climatic Sciences, Makerere University, P. O Box 7062, Kampala, Uganda; 2 Department of Environmental and Global Health, College of Public Health and Health Professions, University of Florida, 2055 Mowry Rd, Gainesville, FL 32610 USA; 3 Emerging Pathogens Institute, University of Florida, Gainesville, FL USA; 4 Wellstar College of Health and Human Services, Kennesaw State University, Kennesaw, NW USA

**Keywords:** Malaria, Children, Household, Risk factor, Refugee, Settlement, Uganda

## Abstract

**Background:**

While 5% of 247 million global malaria cases are reported in Uganda, it is also a top refugee hosting country in Africa, with over 1.36 million refugees. Despite malaria being an emerging challenge for humanitarian response in refugee settlements, little is known about its risk factors. This study aimed to investigate the risk factors for malaria infections among children under 5 years of age in refugee settlements in Uganda.

**Methods:**

We utilized data from Uganda’s Malaria Indicator Survey which was conducted between December 2018 and February 2019 at the peak of malaria season. In this national survey, household level information was obtained using standardized questionnaires and a total of 7787 children under 5 years of age were tested for malaria using mainly the rapid diagnostic test. We focused on 675 malaria tested children under five in refugee settlements located in Yumbe, Arua, Adjumani, Moyo, Lamwo, Kiryadongo, Kyegegwa, Kamwenge and Isingiro districts. The extracted variables included prevalence of malaria, demographic, social-economic and environmental information. Multivariable logistic regression was used to identify and define the malaria associated risk factors.

**Results:**

Overall, malaria prevalence in all refugee settlements across the nine hosting districts was 36.6%. Malaria infections were higher in refugee settlements located in Isingiro (98.7%), Kyegegwa (58.6%) and Arua (57.4%) districts. Several risk factors were significantly associated with acquisition of malaria including fetching water from open water sources [adjusted odds ratio (a*OR*) = 1.22, 95% *CI*: 0.08–0.59, *P* = 0.002], boreholes (a*OR* = 2.11, 95% *CI*: 0.91–4.89, *P* = 0.018) and water tanks (a*OR* = 4.47, 95% *CI*: 1.67–11.9, *P* = 0.002). Other factors included pit-latrines (a*OR* = 1.48, 95% *CI*: 1.03–2.13, *P* = 0.033), open defecation (a*OR* = 3.29, 95% *CI*: 1.54–7.05, *P* = 0.002), lack of insecticide treated bed nets (a*OR* = 1.15, 95% *CI*: 0.43–3.13, *P* = 0.003) and knowledge on the causes of malaria (a*OR* = 1.09, 95% *CI*: 0.79–1.51, *P* = 0.005).

**Conclusions:**

The persistence of the malaria infections were mainly due to open water sources, poor hygiene, and lack of preventive measures that enhanced mosquito survival and infection. Malaria elimination in refugee settlements requires an integrated control approach that combines environmental management with other complementary measures like insecticide treated bed nets, indoor residual spraying and awareness.

**Graphical Abstract:**

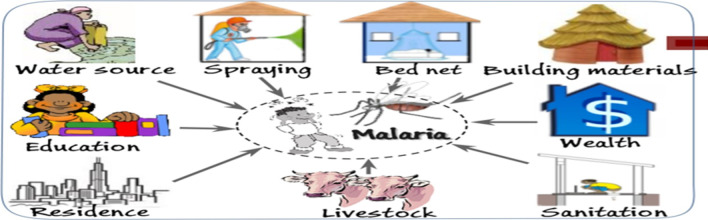

**Supplementary Information:**

The online version contains supplementary material available at 10.1186/s40249-023-01090-3.

## Background

Malaria is among the major global life-threatening diseases which is spread to humans by bites from infected female *Anopheles* mosquitoes [1]. Although six *Plasmodium* parasite species (i.e. *P. falciparum*, *P*. *vivax, P. malariae*, *P. ovale curtisi*, *P. ovale wallikeri* and *P. knowlesi)* are known to cause malaria in humans, *P. falciparum* is the most prevalent malaria parasite in sub-Saharan Africa (SSA), contributing to most of the malaria cases and deaths [2]. By 2021, there was an estimated 234 million malaria cases and 593,000 deaths within the World Health Organization (WHO) African Region [1]. The malaria burden was further amplified by coronavirus disease 2019 (COVID-19) disruptions which constrained malaria chemoprevention, distribution of insecticide-treated bed nets (ITNs), indoor residual spraying (IRS), malaria testing and treatment [3]. By 2021, an estimated additional 13.4 million cases were attributed to the disruptions during the COVID-19 pandemic [1]. In addition, climate change is adding another layer of complication to the burden considering that the transmission frontiers and the risk for malaria have shifted further to the eastern and southern parts of the African continent especially in highlands and densely populated regions [4, 5].

As the world strives to eliminate malaria [1], the plight of refugees, displaced people, and asylum seekers must not be forgotten. It is widely recognised that human mobility influences the spread of infectious diseases including influenza, cholera, malaria, dengue, schistosomiasis and Ebola among others [6–8]. Human mobility is one of determinants of many infectious disease transmission dynamics by either introducing pathogens into susceptible populations or changing the frequency of contacts between infected and susceptible individuals or both [9]. As of late 2021, the United Nations Refugee Agency estimated that globally, there were 89.3 million forcibly displaced people [10], with almost two-thirds of the people affected by humanitarian emergencies inhabiting malaria endemic regions [11].

The high prevalence of malaria among the displaced populations in Africa constitutes an emerging challenge for humanitarian response [12]. Vector borne and other infectious diseases present many challenges in refugee settlements due to inequalities, limited access to healthcare services, and crowded environments which enable rapid disease spread [13]. The risk for malaria infections can increase among refugees especially when immunologically naive individuals with little or no prior malaria exposure move to areas of more intense transmission [11]. Besides, the influx of refugees from endemic countries can be associated with imported malaria [6] which can contribute to secondary transmission and the spread of drug resistance while threatening long-term elimination goals [14].

The population subgroups considered to be at higher risk of contracting malaria include children under 5 years of age [15, 16], pregnant women [17], and patients with HIV/AIDS [18]. Refugees are rendered vulnerable to malaria infections by their lack of protective immunity, increased concentration of people in exposed settings, limited distribution of ITNs, inadequate IRS, insufficient rapid clinical diagnostic and treatment responses [19, 20]. Other risk factors include outdoor night activities, wearing short clothes, residing in unfinished houses, poor drainage [21] and acute malnutrition among children [22].

While 5% of the 247 million global malaria cases are from Uganda [1], the country is also one of the top refugee hosting countries in Africa [23]. Refugee settlements in Uganda are predominantly located in rural areas of hosting districts usually with active malaria transmission. Such areas are typically characterised by overcrowding, inadequate and temporary shelters, limited vector control efforts and poor access to water and sanitation [24]. These conditions make refugee settlements susceptible to high risks of malaria transmission. Elsewhere, it has been documented that malaria is among the leading causes of morbidity and mortality among children under 5 years of age in refugee settlements [16, 21, 25]. Despite these potential risks and challenges, studies on refugees in Uganda have concentrated much on adolescent sexual behaviour [26], psychosocial impact of COVID-19 [27], impact of COVID-19 on food security [28], access to education [29], agroforestry [30], environmental degradation [31] among others, with limited focus on malaria risk factors, treatment and preventive measures [25].

Understanding the key risk factors for malaria infections among children in refugee settlements is crucial for (re)designing humanitarian responses and selecting appropriate intervention strategies for malaria prevention, control, even elimination. This is only possible with adequate research and evidence to support the development of effective and sustainable management strategies. To bridge this knowledge gap, this study utilised data from the 2018–2019 Uganda Malaria Indicator Survey (UMIS) which is the first national wide malaria survey in Uganda to include households and people in refugee settlements [32]. This study aimed at providing an overview the prevalence of malaria infections and local context risk factors for malaria infections among children under 5 years of age in refugee settlements of Uganda.

## Methods

### Study area

**Fig. 1:**
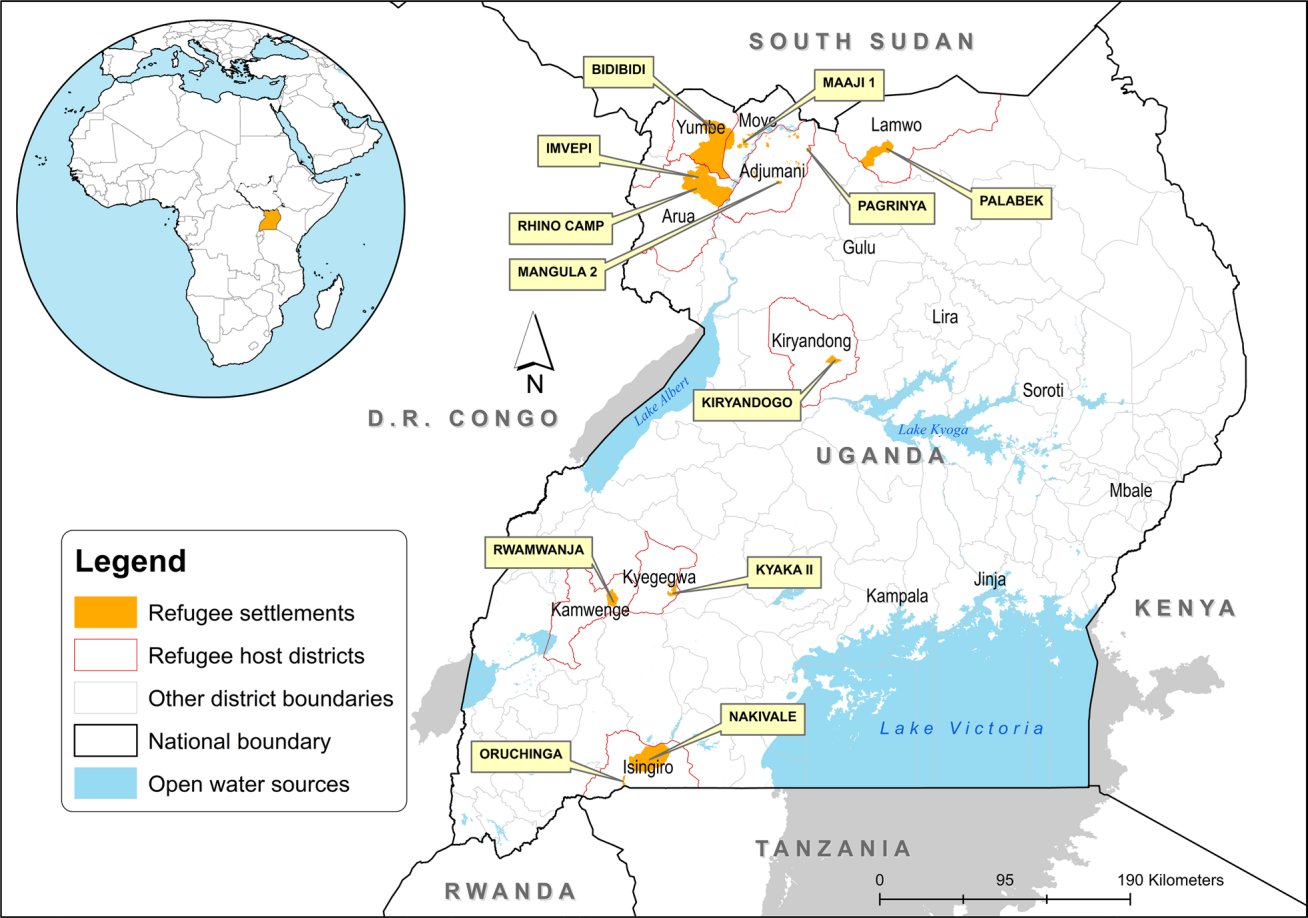
Refugee hosting districts in Uganda

Uganda is the third largest refugee-hosting country in the world after Turkey and Pakistan with over 1.36 million refugees [24]. By 2018, South Sudan made up the largest refugee population in Uganda (985,512), followed by the Democratic Republic of Congo (D.R. Congo) (271,967) and Burundi (36,677) [24]. About 70,988 refugees from Ethiopia, Eritrea, Rwanda, Somalia and Sudan have lived in protracted exile in Uganda for the past three decades [33]. The majority of the refugee population are women and children (82%), with 56% below the age of 15, while 25% are younger than 5 years [34]. This study focused on all refugee settlements located in nine districts of Yumbe, Arua, Adjumani, Moyo, Lamwo, Kiryadongo, Kyegegwa, Kamwenge and Isingiro, as shown in Fig. 1.

### Data source

Datasets for this study were obtained from the 2018–2019 UMIS. To link the demographic, social-economic and environmental variables captured in the 2018–2019 UMIS to malaria infections, we conducted a literature review and the variables deemed relevant for the refugee settlements were identified as shown in  Additional file 1: Table S1. The 2018–2019 UMIS was obtained from the Demographic and Health Surveys program. The 2018–2019 UMIS was the third malaria survey to be conducted in Uganda (after the 2009 UMIS and 2014–2015 UMIS) focusing on refugee settlements and their hosting districts and included information on malaria parasite prevalence, anemia, and status of key malaria indicators [32]. The survey was based on a nationally representative sample of 320 clusters. Standardised questionnaires were designed to collect the demographic, social, economic and environmental information of the surveyed households. Both rapid diagnostic test (RDT) and the blood smear test (BST) were used to test malaria parasitemia among children under 5 years of age with consent from household heads [32].

### Study population, sample size and variable selection

The 2018–2019 UMIS involved a total of 8125 children under 5 years of age of whom, 3481 children were from refugee settlements shown in Fig. 1. In this survey, 7787 children under 5 years of age were tested for malaria country-wide using both RDT and BST methods. In this paper, we only focused on 675 children under 5 years who were tested for malaria in refugee settlements. For quality control, results of the RDT and BST were checked for their completeness. In this study, we used the RDT results because the BST results had significant missing values. The explanatory or independent variables extracted from the survey datasets were those factors which had the ability to potentially influence mosquito survival, biting, feeding, parasite development and breeding (Additional file 1: Table S1). These variables were grouped into risk factors in line with the demographic and social-economic aspects, water, sanitation and housing, malaria prevention practices, knowledge on the causes and prevention of malaria. Variables extracted from the datasets were categorized to perform the analysis. More details are shown in Additional file 1: Table S2.

### Data analysis

Data entry and all the analyses were conducted using JMP software, version 13 (JMP Statistical Discovery LLC, North Carolina, USA). Descriptive analysis was performed on all study variables. Univariate logistic regression was used to estimate the unadjusted odds ratio (uOR) of each independent variable to malaria infection. Variables that were significantly associated with malaria infection with a significance level of *P* < 0.20 were selected for possible inclusion in multivariable logistic regression. We chose *P* < 0.20 as the threshold for including variables in the multivariable model because this has been suggested elsewhere as an appropriate cut-off. In the multivariable logistic regression, we used a backward stepwise strategy to estimate the adjusted odds ratios (a*ORs*) and corresponding 95% confidence intervals (*CI*) of the household variables in association with malaria infections with a significance level of *P* < 0.05. Results of both the descriptive and logistic regression analyses were displayed in a table format.

## Results

### Prevalence of malaria infections among children in refugee settlements

A total of 675 children below the age of five was used in this study, of which 29.6% were aged 0–15 months, 21.3% aged 16–30 months, 24.9% aged 31–45 months and 24.1% age 46–60 months. Overall, the prevalence of malaria infections in all refugee settlements across the nine hosting districts was 36.6%. Malaria infections were higher in refugee settlements located in Isingiro (98.7%), Kyegegwa (58.6%) and Arua (57.4%) districts. The prevalence of malaria was low in refugee settlements located in Adjumani (19.2%) and Kiryandongo (7.7%) districts.

### Determinants of malaria infections among children in refugee settlements

Table 1 depicts the demographic and social-economic risk factors associated with malaria infections. Indeed, significant risk factors included children’s age, age of household head, household wealth status, mother’s level of education, and type of cooking fuel. The results suggest that the odds of contracting malaria were significantly higher in refugee households whose children were aged 31–45 months (a*OR* = 2.14, 95% *CI*: 1.32–3.47) and above 45 months (a*OR* = 2.01, 95% *CI*: 1.22–3.32) compared to those households whose children were under 31 months. Households whose heads were aged between 15 and 24 years were 1.74 times (a*OR* = 1.74, 95% *CI*: 1.08–2.79) more likely to have their children getting malaria infections than those whose heads were 25 years and above. Refugee households with mothers with no education were 1.35 times (aOR = 1.35, 95% CI 0.92–1.96) more likely to have their children contracting malaria compared to households with educated mothers. Poor households were 4.23 times (a*OR* = 4.23, 95% *CI*: 1.19–14.9) more likely to have their children at risk of malaria infections compared to rich households. Households which used firewood and straw for cooking were 2.20 (a*OR* = 2.20, 95% *CI*: 1.17–4.15) and 2.51 (a*OR* = 2.51, 95% *CI*: 0.68–9.25) times more likely to have their children contracting malaria respectively compared to households which used charcoal for cooking (*P* < 0.05).

Further, Table 1 shows environmental malaria risk factors. Households whose main sources of domestic water were open water sources, boreholes and water tanks, were 1.22 (a*OR* = 1.22, 95% *CI*: 0.08–0.59), 2.11 (a*OR* = 2.11, 95% *CI*: 0.91–4.89) and 4.47 (a*OR* = 4.47, 95% *CI*: 1.67–11.9) times more likely to have their children contracting malaria respectively, compared to households which fetched water from public water taps (*P* < 0.05). Walk time distance to water sources of 0–15 min was associated with significantly higher odds of getting malaria among children (a*OR* = 1.68, 95% *CI*: 1.11–2.56) compared to walk time distance above 15 min. Households which used pit-latrines with slabs and those without any toilet facility or used bushes were 1.48 (a*OR* = 1.48, 95%* CI*: 1.03–2.13) and 3.29 (a*OR* = 3.29, 95% *CI*: 1.54–7.05) times more likely to have their children obtaining malaria (*P* < 0.05). Using open pits also increased the risk for malaria infections (a*OR* = 6.67, 95%* CI*: 0.47–0.97). From Table 1, it is further indicated that households whose walls were constructed using thatch and cardboard were 4.50 (a*OR* = 2.50, 95% *CI*: 0.11–56.0) and 2.30 (a*OR* = 2.30, 95% *CI*: 1.39–13.2) times more likely to have their children contracting malaria compared to households whose walls were built with brick and cement or mud (*P* < 0.05). Additionally, households with thatch roofs were 2.12 (a*OR* = 2.12, 95%* CI*: 0.94–4.79) times more likely to have their children contracting malaria compared households with roofs constructed of tarpaulins and iron sheets.

From Table 1, malaria prevention risk factors are presented. It is clearly indicated that households which did not have ITNs or sprayed their households with insecticides, were 1.15 (a*OR* = 1.15, 95% *CI*: 0.43–3.13) and 8.04 (a*OR* = 8.04, 95% *CI:* 2.47–26.2) times more likely to have their children contracting malaria compared to households which had implemented these preventive interventions. Further, households whose children did not sleep under ITNs were associated with significantly higher risk of malaria infection in children (a*OR* = 1.30, 95% *CI*: 0.58–2.49) compared to those households whose children slept under ITNs. Households which did not have access to malaria preventive medicine were 4.84 (a*OR* = 4.84, 95% *CI*: 1.82–12.8) more likely to have their children getting malaria compared to those households which had access.

Risk factors associated with knowledge on malaria transmission and prevention are also presented in Table 1. Households which did not know that mosquito bites caused malaria were 1.09 (a*OR* = 1.09, 95% *CI*: 0.79–1.51) times more likely to have their children contracting malaria compared to those which knew. Households which indicated that eating maize and mangoes as the causes of malaria were 2.96 (a*OR* = 2.96, 95% *CI*: 1.13–7.75) and 1.22 (a*OR *= 1.22, 95%* CI*: 0.47–3.19) times more likely to have their children contracting malaria respectively compared to those households which knew the actual causes (*P* < 0.05). Households which indicated that malaria could not be avoided were 2.14 (a*OR *= 2.14, 95% *CI*: 1.29–3.55) times more likely to have their children contracting malaria. The odds of children contracting malaria were high in households which did not know that sleeping under the net (a*OR* = 1.61, 95% *CI*: 1.16–2.23) and destroying breeding sites (a*OR* = 1.99, 95% *CI*: 1.44–2.76) were the ways of avoiding malaria.

**Table 1:** Risk factors for malaria infections among children under five in refugee settlements located in Uganda

Risk factors (*n* = 675)	Total *n* (%)	Negative *n* (%)	Positive *n* (%)	u*OR* (95% *CI*)	*P*-value	a*OR* (95% *CI*)	*P*-value
*Demographic and socio-economic risk factors*							
Age of child, months							
0–15	200 (29.6)	151 (75.5)	49 (24.5)	0.46 (0.29–0.73)	0.0008*	0.49 (0.30–0.82)	0.0061*
16–30	144 (21.3)	104 (72.2)	40 (27.8)	0.55 (0.34–0.89)	0.0150*	0.59 (0.34–1.01)	0.0462*
31–45	168 (24.9)	97 (57.7)	71 (42.3)	2.26 (1.45–5.52)	0.0003*	2.14 (1.32–3.47)	0.0021*
Above 45	163 (24.1)	96 (58.9)	67 (41.1)	2.15 (1.37–3.37)	0.0008*	2.01 (1.22–3.32)	0.0061*
Age of household head, years							
15–24	90 (13.3)	62 (68.9)	28 (31.1)	2.06 (1.63–2.61)	0.0001**	1.74 (1.08–2.79)	0.0219*
25–34	312 (46.2)	210 (67.3)	102 (32.7)	0.49 (0.38–0.62)	0.0001**	0.57 (0.36–0.92)	0.0219*
35–44	157 (23.3)	97 (61.8)	60 (38.2)	0.62 (0.45–0.85)	0.0003*	0.68 (0.39–1.17)	0.1660
45 and above	116 (17.2)	79 (68.1))	37 (31.9)	0.76 (0.46–1.26)	0.2813	–	–
Sex of household head							
Male	268 (39.7)	166 (61.9)	102 (38.1)	1.7 (1.28–2.36)	0.0001**	1.45 (0.98–2.12)	0.0571
Female	407 (60.3)	282 (69.3)	125 (30.7)	0.57 (0.42–0.78)	0.0001**	0.69 (0.47–1.01)	0.0571
Mother’s educational level							
No education	355 (52.6)	230 (64.8)	125 (35.2)	2.75 (1.42–5.32)	0.0003*	1.35 (0.92–1.96)	0.0205*
Primary level	264 (39.1)	178(67.4)	86 (32.6)	0.48 (0.37–0.62)	0.0001**	0.74 (0.51–1.08)	0.1116
Ordinary level	45 (6.7)	33 (73.3)	12 (26.7)	0.36 (0.19–0.70)	0.0002*	0.49 (0.65–3.43)	0.3483
Advanced level	11 (1.6)	7 (63.6)	4 (36.4)	0.20 (0.18–22.11)	0.5714	–	–
Number of household members							
1–5	197 (29.2)	133 (67.5)	64 (32.5)	1.9 (1.53–2.34)	0.0001**	1.04 (0.68–1.60)	0.8297
6–10	382 (56.6)	250 (65.4)	132 (34.6)	0.53 (0.43–0.65)	0.0001**	0.72 (0.40–1.30)	0.2802
Above 10	96 (14.2)	65 (67.7)	31 (32.3)	0.48 (0.31–0.73)	0.0001**	1.38 (0.77–2.49)	0.3949
Household wealth status							
Poor	639 (94.7)	418 (65.4)	221 (34.6)	3.33 (1.34–8.30)	0.0097*	4.23 (1.19–14.94)	0.0251*
Rich	36 (5.3)	30 (83.3)	6 (16.7)	0.3 (0.12–0.75)	0.0097*	0.24 (0.06–0.83)	0.0251*
Owned livestock, herds							
No	443 (65.6)	294 (66.4)	149 (33.6)	0.66 (0.49–0.89)	0.0008*	0.72 (0.49–1.07)	0.1128
Yes	232 (34.4)	154 (66.4)	78 (33.6)	1.51 (1.11–2.04)	0.0008*	1.38 (0.93–2.05)	0.1128
Type of cooking fuel used							
Charcoal	97 (14.4)	79 (81.4)	18 (18.6)	0.54 (0.33–0.88)	0.0127*	0.18 (0.04–0.81)	0.0250*
Firewood	571 (84.6)	362 (63.4)	209 (36.6)	1.85 (1.14–3.00)	0.0113*	2.20 (1.17–4.15)	0.0143*
Straw/grass	7 (1.0)	3 (42.9)	4 (57.1)	8.43 (4.83–14.73)	0.0001**	2.51 (0.68–9.25)	0.0250*
*Environmental malaria risk factors*							
Source of drinking water							
Open water sources	37 (5.5)	29 (78.4)	8 (21.6)	1.86 (1.01–3.22)	0.0044*	1.22 (0.08–0.59)	0.0029*
Boreholes	293 (43.4)	199 (66.8)	99 (33.2)	0.55 (0.31–0.98)	0.0046*	2.11 (0.91–4.89)	0.0189*
Public water taps	290 (43.0)	196 (67.6)	94 (32.4)	0.19 (0.11–0.35)	0.0001**	0.36 (1.01–5.53)	0.0482*
Water tanks	55 (8.1)	24 (48.0)	26 (52.0)	5.03 (2.83–8.92)	0.0001**	4.47 (1.67–11.93)	0.0029*
Walk time distance to water sources							
0–15 min	337 (49.9)	232 (68.8)	105 (31.2)	2.10 (1.54–2.88)	0.0001**	1.68 (1.11–2.56)	0.0149*
16–30 min	180 (26.7)	122 (67.8)	58 (32.2)	0.47 (0.35–0.65)	0.0001**	0.77 (0.48–1.23)	0.0817*
Above 30 min	158 (23.4)	94 (59.5)	64 (40.5)	0.68 (0.49–0.94)	0.0018*	0.59 (0.39–0.90)	0.0149*
Type of toilet facility used							
Pit-latrines with slabs	372 (55.1)	236 (63.4)	136 (36.6)	1.44 (1.03–2.01)	0.0305*	1.48 (1.03–2.13)	0.0332*
Open pits	270 (40.0)	196 (67.6)	94 (32.4)	8.03 (4.77–13.54)	0.0001**	6.67 (0.47–0.97)	0.0332*
No toilet/Open defecation	33 (4.9)	16 (48.5)	17 (51.5)	5.58 (3.52–8.83)	0.0001**	3.29 (1.54–7.05)	0.0021*
Main floor materials							
Earth floors	533 (79.0)	345 (64.7)	188 35.3)	0.30 (0.19–0.47)	0.0001**	0.65 (0.33–1.29)	0.2197
Dung floors	110 (16.3)	77 (70.0)	33 (30.0)	0.38 (0.19–0.76)	0.0057*	0.86 (0.19–3.75)	0.8367
Cement floors	32 (4.7)	26 (81.3)	6 (18.8)	3.32 (2.11–5.23)	0.0001**	1.79 (0.48–6.68)	0.3873
Main wall materials							
Thatch walls	243 (36.0)	153 (63.5)	88 (36.5)	9.21 (2.44–34.70)	0.0001**	4.50 (0.11–56.00)	0.0109*
Cardboard walls	16 (2.4)	7 (43.8)	9 (56.3)	3.09 (1.13–8.42)	0.0024*	2.30 (1.39–13.22)	0.0386*
Bricks with cement walls	16 (2.4)	12 (75.0)	4 (25.0)	0.11 (0.02–0.88)	0.0029*	0.39 (0.07–8.93)	0.5626
Bricks with mud walls	400 (59.3)	274 (68.5)	126 (31.5)	0.26 (0.09–1.07)	0.0017*	0.64 (0.10–7.83)	0.6374
Main roofing materials							
Thatch roofs	420 (62.2)	264 (62.9)	156 (37.1)	1.47 (1.03–2.09)	0.0033*	2.12 (0.94–4.79)	0.0007*
Tarpaulin roofs	147 (21.8)	99 (67.3)	48 (32.7)	0.68 (0.48–0.97)	0.0037*	0.47 (0.21–1.07)	0.0007*
Iron sheet roofs	108 (16.0)	85 (78.7)	23 (21.3)	0.87 (0.53–1.42)	0.5756	–	–
*Malaria prevention risk factors*							
Used mosquito bed-nets							
No	103 (15.3)	63 (61.2)	40 (38.8)	2.89 (2.11–3.95)	0.0001**	1.15 (0.43–3.13)	0.0031*
Yes	572 (84.7)	385 (67.3)	187 (32.7)	0.35 (0.25–0.47)	0.0001**	0.86 (0.32–2.35)	0.0031*
Sprayed against mosquitoes							
No	673 (99.7)	446 (66.3)	227 (33.7)	3.79 (2.76–5.23)	0.0001**	8.04 (2.47–26.21)	0.0005*
Yes	2 (0.3)	2 (100.0)	0 (0.0)	0.26 (0.19–0.36)	0.0001**	0.12 (0.04–0.40)	0.0005*
Children slept under ITN							
No	169 (25.0)	108 (63.9)	61 (36.1)	1.76 (1.24–2.50)	0.0017*	1.30 (0.58–2.49)	0.0451*
All children	376 (55.7)	257 (68.4)	119 (31.6)	0.57 (0.39–0.81)	0.0017*	0.77 (0.34–1.73)	0.0421*
Some children	127 (18.8)	82 (64.6)	45 (35.4)	2.12 (1.46–3.09)	0.0001**	1.33 (0.69–2.57)	0.3855
Had access to malaria medicine							
No	635 (94.1)	412 (64.9)	223 (35.1)	5.88 (2.03–17.02)	0.0011*	4.84 (1.82–12.84)	0.0016*
Yes	40 (5.9)	36 (90.0)	4 (10.0)	0.17 (0.06–0.49)	0.0011*	0.21 (0.08–0.55)	0.0016*
*Knowledge on the causes of malaria*							
Mosquito bites							
No	520 (77.0)	347 (66.7)	173 (33.3)	1.45 (1.07–1.97)	0.0155*	1.09 (0.79–1.51)	0.0051*
Yes	155 (23.0)	101 (65.2)	54 (34.8)	0.69 (0.51–0.93)	0.0155*	0.91 (0.66–1.26)	0.0051*
Eating maize							
No	666 (98.7)	441 (66.2)	225 (33.8)	0.27 (0.19–0.38)	0.0001**	0.34 (0.13–0.89)	0.0276*
Yes	9 (1.3)	7 (77.8)	2 (22.2)	3.65(2.65–5.00)	0.0001**	2.96 (1.13–7.75)	0.0276*
Eating mangoes							
No	660 (97.8)	436 (66.1)	224 (33.9)	0.29 (0.21–0.39)	0.0001**	0.82 (0.31–2.14)	0.0483*
Yes	15 (2.2)	12 (80.0)	3 (20.0)	3.46 (2.52–4.75)	0.0001**	1.22 (0.47–3.19)	0.0438*
Stagnant water							
No	639 (94.7)	422 (66.0)	217 (34.0)	1.33 (0.63–2.82)	0.4464	–	–
Yes	36 (5.3)	26 (72.2)	10 (27.8)	0.75 (0.35–1.58)	0.4464	–	–
Poor hygiene						–	–
No	613 (90.8)	405 (66.1)	208 (33.9)	1.16 (0.66–2.04)	0.6020	–	–
Yes	62 (9.2)	43 (69.4)	19 (30.6)	0.86 (0.48–1.51)	0.6020	–	–
Not sleeping under ITNs						–	–
No	639 (94.7)	424 (66.4)	215 (33.6)	1.01 (0.49–2.07)	0.9691	–	–
Yes	36 (5.3)	24 (66.7)	12 (33.3)	0.99 (0.48–2.00)	0.9691	–	–
*Knowledge on ways to avoid malaria*							
Malaria can be avoided							
No	99 (14.7)	64 (64.6)	35 (35.4)	2.68 (1.96–3.66)	0.0001**	2.14 (1.29–3.55)	0.0031*
Yes	576 (85.3)	384 (66.7)	192 (33.3)	0.37 (0.27–0.51)	0.0001**	0.47 (0.28–0.77)	0.0031*
Sleeping under ITNs							
No	619 (91.7)	412 (66.6)	207 (33.4)	1.99 (1.47–2.70)	0.0001**	1.61 (1.16–2.23)	0.0038*
Yes	56 (8.3)	36 (64.3)	20 (35.7)	0.50 (0.37–0.68)	0.0001**	0.62 (0.45–0.86)	0.0038*
Using mosquito repellents							
No	672 (99.6)	446 (66.4)	226 (33.6)	1.01 (0.09–11.24)	0.9913	–	–
Yes	3 (0.4)	2 (66.7)	1 (33.3)	0.99 (0.08–10.90)	0.9913	–	–
Spraying with insecticides							
No	665 (98.5)	439 9 (66.0)	226 (34.0)	4.63 (0.59–36.83)	0.1470	–	–
Yes	10 (1.5)	9 (90.0)	1 (10.0)	0.22 (0.02–1.71)	0.1470	–	–
Destroying breeding sites							
No	610 (90.4)	395 (64.8)	215 (35.2)	2.40 (1.26–4.59)	0.0080*	1.99 (1.44–2.76)	0.0001*
Yes	65 (9.6)	53 (81.5	12 (18.5)	0.42 (0.22–0.79)	0.0080*	0.50 (0.36–0.69)	0.0001*

*uOR* Unadjusted odd ratio, *aOR* Adjusted odd ratio, *ITNs* Insecticide-treated bed-nets. *Statistical significance at *P*-value < 0.05; ** high statistical significance at *P*-value < 0.01. The en-dashes (–) indicate that the predictors were excluded from the multivariable regressions. All *P*-values are within the limits of 95% confidence interval (*CI*)

## Discussion

The concentration and crowding of refugees in settlements in Uganda is a fertile ground for malaria transmission within these settings and hosting communities. The risk factors discussed below relate to the survival, biting, feeding, parasite development and breeding of mosquitoes.

### Demographic and socio-economic risk factors

#### Age

Although previous studies indicated that infants less than 12 months of age were the most vulnerable groups to malaria infections [35–37], the results of this study suggested that households with children aged 31 months and above were more likely to have their children contracting malaria in refugee settlements. There are several potential reasons for the difference. Refugee settlements are distinct and complex with people coming from diverse social-cultural and economic backgrounds. Thus, little is known of the neonatal and infant care practices, social norms, and behavioral patterns adopted by refugees to reduce malaria infections and other diseases. The high risk of malaria infection in children aged 31 months and older could, however, be attributed to the fact that at this age, the children were more active (i.e., crawling, walking, removing clothes, uncovering during sleep, etc.), making them more susceptible to mosquito bites. Moreover, during this age, most of children are weaned when they have not yet acquired natural immunity to manage or fight high parasite density [38]. Other studies have linked malaria risk among children between 24 and 60 months to malnutrition, although the evidence remains inconclusive [39]. The shift in age groups at high risk of malaria as observed from children aged 31 months and above in this study, suggests the need to expand prevention and free treatment strategies for older children in order to cover the peak transmission months in refugee settlements.

The odds for contracting malaria among children were significantly higher among households headed by refugees aged 15–24 years. This is due to the fact that refugees aged 15–24 years were within the category of ‘child and young migrants’ and may not have had adequate knowledge about child care, malaria transmission and prevention and at the same time, may have lacked financial resources and support for malaria prevention measures and treatment. This study further revealed that increase in the age of household head reduced the odds of malaria infections in children. This result is contrary to other studies conducted in Nigeria [40] and SSA which indicate that increase in ages of both male and female heads of households increased the odds of malaria prevalence among children under 5 years of age [41, 42]. This contradiction can be explained by the population variations and age dynamics between refugee and non-refugee settlements. Moreover, such studies never considered refugee settlements yet context specific behavioural and social cultural information are key to understanding the interactions that propel the risks for malaria within these local dynamic settings.

#### Mother’s educational level

The result of this study shows that lack of education among refugee mothers was significantly associated with a risk of malaria among children compared to those households with educated mothers. This result is consistent with findings from other studies [21, 40–42]. Since mothers are at the core of family welfare and wellbeing, their level of education is an essential social determinant of health and influences their ability to make better decisions about the health of children. Since adult literacy programmes for mothers in refugee settlements are rare, larger scale health promotional campaigns focusing on malaria diagnosis, prevention, and treatment are required. Such health education campaigns have worked well in endemic communities where knowledge about malaria and newer interventions are often lacking [43]. For example, a study conducted in Ethiopia indicated that children whose parents received training and awareness about use of ITNs were found to be at a lower risk of malaria infections compared to those who did not have any training [42].

#### Household wealth status

The findings of this study further suggest that poor refugee households were more likely to have their children contracting malaria. This result is in line with other study findings [36, 43]. The cost of malaria diagnosis, treatment and prevention may seem high for the poor households who face other constraints and daily expenses. Distribution of ITN, conducting IRS and destruction of mosquito breeding sites are options that need to be explored in refugee settlements.

#### Type of cooking fuel used

Although smoke from biomass cooking is often associated with reduced mosquito abundance and malaria transmission [44], the results of this study indicated that households which used firewood and straw for cooking were more likely to have their children contracting malaria compared to households which used charcoal. This result is consistent with other studies [45]. There are potential reasons to this finding. Women and children in refugee settlements gather much of the firewood used in their households and this exposes them to frequent mosquito bites in the forests and bushes where firewood is obtained. Besides, firewood piles near households serve as daytime hiding place for mosquitoes. Additionally, the shift in mosquito behavior from indoor late-night biting to outdoor early-evening biting [46, 47] coincides well with the major outdoor cooking activities in most refugee homesteads. Some studies in non-refugee settlements, however, indicate no association between outdoor cooking practices and malaria infections among children [48]. Nevertheless, epidemiological modelling [13] is required to better understand the relationships between cooking practices, cooking fuel emissions, mosquito activity and risk of malaria acquisition among children in refugee settlements. Such modelling can be vital in supporting appropriate malaria prevention messaging and evidence-based decision making in this context. Although charcoal use was associated with reduced malaria risk, it is one of the fundamental drivers of deforestation in refugee hosting districts and beyond, thus, its use in malaria control programs is not advisable in both short and long run.

### Environmental malaria risk factors

#### Sources of drinking water

This study found out that refugee households which obtained water from open water sources and water tanks were more likely to have their children contracting malaria. This result is consistent with other studies [41–43]. This is likely due to several reasons. First, open water sources like rivers, ponds, lakes, swamps, dams, wells and springs serve as meeting places for humans and mosquitoes. Given the fact that refuge mothers and children fetch most of the water from these sources, the chances of mosquito bites are high. Second, these water sources are potential oviposition sites which are crucial for reproductive success and population dynamics of mosquitoes [49]. Third, these open water sources shorten the gonotrophic cycle [50] especially when located near refugee households. Water tanks as a key malaria risk factor is not surprising because they act as breeding sites for mosquitoes. Households which obtained water from boreholes and public water taps were less likely to have their children contracting malaria, although other studies indicated the opposite [15, 51].

#### Walk time distance to water sources

This study also revealed short walk time distance (0–15 min) to water sources was one of the risk factors for malaria among children. This result is in line with other studies focusing on malaria infections and distance to water sources [52, 53]. Although distant water sources were associated with reduced malaria risk, this result should not hamper efforts geared to improve water access in refugee settlements. This is because reducing the time to fetch water has been observed to improve child health [54]. Thus, given the land use and land cover changes within refugee settlements, environmental management practices such as draining stagnant water, dredging water channels, clearing vegetation among others, should be applied to water sources to reduce mosquitoes breeding and survival.

#### Household sanitation

Refugee households which had pit-latrines and those without any toilet facility were more likely to have their children contracting malaria. This result is not surprising because mosquitoes have overtime started changing their breeding preference to contaminated surroundings [55]. Similar findings have been observed in other studies [21, 41, 51, 53]. Based on this finding, interventions need to be strengthened so that pit-latrines which are commonly used within refugee settlements are vector borne free. For example, mosquitoes in pit-latrines can be suppressed when expanded polystyrene beads are used.

#### Building materials

This study revealed that the odds of malaria infections increased among children living in houses with grass thatch roofs, thatch and cardboard walls. This result is consistent with a systematic review and meta-analysis study on socioeconomic determinants of malaria burden in SSA [42]. Walls made up with thatch and cardboards allow mosquitoes to enter into households with ease [56]. Additionally, thatch provides conducive indoor resting grounds for mosquitoes since there are associated with cool temperatures which can sustain survival of mosquitoes indoors [57]. Thus, the choice of building materials for house construction in refugee settlements should be carefully selected to minimize malaria risk. For example, this study revealed that households with brick walls and iron sheet roofs were less likely to have their children contracting malaria, although the odds were not significant. However, a recent study conducted in SSA [58] revealed that metal-roofed houses contributed to the decline in malaria burden, since they were associated with higher temperatures and lower humidity which reduced survivorship of indoor-resting mosquitoes.

### Malaria prevention risk factors

The odds of contracting malaria among children were significantly higher in refugee households which did not have ITNs, sprayed against mosquitoes or lacked access to malaria prevention medicines. Some studies reported similar results [21, 40, 42]. Although our study indicated that ITNs and IRS were effective in reducing the risk for malaria, there are limited in scope and efficacy. For example, IRS interventions apply to mosquitoes which feed and rest indoors, while ITNs prevent night mosquito bites just around the beds. This limitation provides opportunities for outdoor active mosquitoes to multiply while sustaining some level of transmission beyond the reach of ITNs and IRS. Additionally, in some cases, mosquitoes have developed resistance to pyrethroids used in ITNs and IRS [59]. Moreover, their continued use has led to an apparent shift in mosquito behavioural traits (i.e., insecticide avoidance and early-exit behaviours among indoor-feeding vectors) [60]. Despite these protection gaps, promotion of ITNs and IRS should continue in refugee settlements. Moreover, since mothers in refugee settlements engage in various outdoor activities at night [21], provision of nets for outdoor spaces in combination with IRS may be an additional strategy to effectively reduce the incidence of malaria among children. The effectiveness and efficacy of ITNs and IRS can be improved and strengthened by promoting environmentally based interventions which reduce mosquito survival and human-vector contact. However, there is a need for continued formative research and strong collaboration between the scientific community and other stakeholders to coordinate malaria elimination strategies that are adapted to the local social context of refugee settlements.

### Knowledge on the causes of malaria

The results of this study suggest that refugee households which did not know the causes and prevention of malaria were more likely to have their children contracting malaria. This result is consistent with a systematic review on studies conducted in Southeast Asia where it was observed that poor knowledge and awareness about malaria transmission were related to some families using nets for reasons other than malaria prevention, such as fishing and for warmth [43]. Similar results are reported from SSA especially among the rural and uneducated individuals [42, 61, 62]. The finding of our study underscores the need for more education, training and communication initiatives to complement delivery of integrated malaria programmes that include mass malaria drug administration in refugee settlements.

## Strength and limitations of the study

The utilization of nationally representative data with a high sample size was the study’s main strength. Thus, our results can be used in generalisation to understand malaria risk factors in other refugee settlements with similar demographic and social-economic settings. However, the study had some limitations. We were not able to include other environmental risk factors like rainfall and temperature, since we used secondary data. Finally, the cross-sectional nature of the study design did not allow a causal-effect relationship to be established with certainty among the identified household level risk predictors and malaria infections. Despite these limitations, the study was obviously sufficiently powered to detect several important risk factors for malaria infections which could be given priority in refugee settlements.

## Conclusions

Malaria infections among children continue to circulate in many refugee settlements in Uganda. This study identified several malaria risk factors which need special attention within the framework of humanitarian assistance. Refugee households which used firewood and straw for cooking, fetched water from open water sources and water tanks were more likely to have their children contracting malaria. In addition, households which used pit-latrines and open defecation were more likely to have their children getting malaria infections. Malaria among children also increased in households with thatch walls and roofs, and those without preventive measures. To sum up, malaria elimination in refugee settlements requires an integrated control approach that combines environmental management with other complementary measures like insecticide treated bed nets, indoor residual spraying and awareness. Future studies can examine the impact of additional factors on the risk for malaria in refugee settlements that have not been studied here.

## Supplementary Information

**Additional file 1.** **Table S1.** Household level risk factors associated with malaria infections among children. **Table S2.** Selected explanatory variables that were used to predict malaria infections.

## Data Availability

The data used in this study can be obtained by sending a request via the DHS Program website and upon approval data can be obtained from https://dhsprogram.com/data/dataset/Uganda_MIS_2018.cfm?flag=1.

## Abbreviations

a*OR*: Adjusted odds ratio
BST: Blood Smear Test
*CI*: Confidence interval
COVID-19: Coronavirus disease 2019
DHS: Demographic and Health Surveys
IRS: Indoor Residual Spraying
ITNs: Insecticide-treated bed-nets
u*OR*: Unadjusted odds ratio
RDT: Rapid Diagnostic Test
UMIS: Uganda Malaria Indicator Survey
WHO: World Health Organization
