# Supplementary material for: Determinants of malaria infections among children in refugee settlements in Uganda during 2018–2019
Source: Infect Dis Poverty. 2023 Apr 10;12:31. doi: 10.1186/s40249-023-01090-3 (PMC10084630; doi:10.1186/s40249-023-01090-3)
Supplement: Supplementary file 1 — Additional file 1. Table S1. Household level risk factors associated with malaria infections among children. Table S2. Selected explanatory variables that were used to predict malaria infections. [file 40249_2023_1090_MOESM1_ESM.docx]

**Determinants of malaria infections among children in refugee settlements in Uganda during 2018–2019**

Henry Musoke Semakula^1,2,a^**_,_* Song Liang*^2,3,^*^b^ Paul Isolo Mukwaya^1,c^, Frank Mugagga^1,d^, Monica Swahn^4,e^, Denis Nseka^1f^, Hannington Wasswa^1,g^, Patrick Kayima^1,h^

^1^ Department of Geography, Geo-informatics and Climatic Sciences, Makerere University, P.O Box 7062, Kampala, Uganda

^2^Department of Environmental and Global Health, College of Public Health and Health Professions, University of Florida, 2055 Mowry Rd Gainesville FL 32610, USA

^3^Emerging Pathogens Institute, University of Florida, Gainesville, FL, USA

^4^Wellstar College of Health and Human Services, Kennesaw State University, NW, USA

^a^Tel. (+256) 779-846-203; (+1)352-709 9310; E-mail: [henry.semakula@mak.ac.ug](mailto:henry.semakula@mak.ac.ug), semhm2000@yahoo.co.uk

^b^Tel. (+1) [352 273-9203](mailto:352%20273-9203); E-mail: songliang@ufl.edu

^c^Tel. (+256) 708-470-320; E-mail: [paul.isolomukwaya@mak.ac.ug](mailto:paul.isolomukwaya@mak.ac.ug), pmukwaya@gmail.com

^d^Tel. (+256) 772-968-421; E-mail: [frank.mugagga@mak.ac.ug](mailto:frank.mugagga@mak.ac.ug), fmugagga@gmail.com

^e^Tel. (+1) 470-578-6962; E-mail: [mswahn@kennesaw.edu](mailto:mswahn@kennesaw.edu)

^f^Tel. (+256) 782-462-298: E-mail: denisnseka1@gmail.com

^g^Tel. (+256) 782-459-399: E-mail: wasswahans@gmail.com

^h^ Tel. (+256) 706-037-721: E-mail: kayimapatrick1789@gmail.com

^*^Correspondence author. Tel. (+256) 779-846-203; (+1) 352-709 9310; E-mail: [henry.semakula@mak.ac.ug](mailto:henry.semakula@mak.ac.ug), semhm2000@yahoo.co.uk

***Review of existing knowledge on malaria risk factors***

To link the demographic and social-economic variables captured in the 2018–19 UMIS to malaria infections, a literature review was necessary. In this study, a comprehensive survey of relevant literature on household level malaria risk factors was conducted. The search for literature focused on review papers and scientific research articles. The search was done in four databases namely MEDLINE (Pubmed®), EMBASE®, Scopus®, and Web of Science®. Google scholar and Mendeley search engines were also used to obtain relevant literature. In both the databases and search engines, keywords and combination of key words [i.e. malaria and social economic factors, malaria and demographic characteristics, malaria risk factors etc.] were used to guide the literature search. The search was limited to literature published between 2018 and 2022. Ten publications, mainly review articles, were identified and reviewed for the documented risk factors associated with malaria infections among children at the household level. Explanatory variables identified from the review and deemed relevant for the refugee settlements are summarized in Table S1.

**Table S1** Household level risk factors associated with malaria infections among children

| **Explanatory variables** | **Description** | **Relationships with malaria infections** | **Sources** |
| --- | --- | --- | --- |
| **S**ex of household head | Determines the ability to obtain malaria treatment and prevention measures | **+** | [1–3] |
| Mother's education | Determines acquisition and usage of malaria prevention methods and treatment | **+** | [1, 3–5] |
| Number of household members | Provides adequate source of blood meal for mosquitos. Larger households are at a higher risk | **+** | [1, 3, 4] |
| Household wealth status | Influences housing quality, ownership and payment for malaria prevention and treatment | **+** | [1, 3–5] |
| Insecticide treated nets (ITNs) | Prevents night mosquito bites around the beds | **+** | [1–4, 6, 7] |
| Indoor residual spraying (IRS) | Kills or repels mosquitoes which feed and rest indoors | **+** | [1–4, 6, 7] |
| Wall materials | Influences the level at which mosquitoes enter households | **+** | [1, 3–5, 7–9] |
| Roof materials | Determines the suitability of indoor resting sites for mosquitoes | **+** | [1, 3–5, 7–9] |
| Floor materials | Influences indoor mosquito density | **+** | [1, 3–5, 7–9] |
| Sanitation | Determines the nature of breeding sites for mosquitoes around households | **+** | [1, 3, 4, 8] |
| Drinking water sources | Influences the nature of ovi-position sites and the time required by vectors to locate them. | **+** | [1, 3, 4, 8] |
| Distance to water sources | Determines the time taken by mosquitoes to find a suitable breeding site. | **+** | [1, 3, 8] |
| Type of cooking fuel | Influences indoor mosquito density, survival and biting rates | **+** | [3, 8] |
| Knowledge of the cause and prevention of malaria | Influences the likelihood of ITN use and malaria treatment | **+** | [10] |

**Table S2.** Selected explanatory variables that were used to predict malaria infections

| **Independent/ explanatory variables** | **Categories** |
| --- | --- |
| ***Socio- Demographic risk factors*** | |
| Age of child, months | 1. 0–15 2. 16–30 3. 31–45 4. Above 45 |
| Age of head of household, years | 1. 15–24 2. 25–34 3. 35–44 4. 45 and above |
| Sex of household head | 1. Male 2. Female |
| Mother's educational level | 1. No education 2. Primary 3. Ordinary level 4. Advanced level 5. Tertiary 6. University |
| Number of household members | 1. 1–5 2. 6–10 3. Above 10 |
| Household wealth | 1. Poor 2. Medium 3. Rich |
| Owns livestock, herds | 1. No 2. Yes |
| Type of cooking fuel used | 1. Charcoal 2. Firewood 3. Straw/grass |
| Sources of drinking water | 1. Open water sources 2. Boreholes 3. Public water taps 4. Tank water |
| ***Environmental malaria risk factors*** | |
| Time to get to water source | 1. 0–15 minutes 2. 16–30 minutes 3. Above 30 minutes |
| Type of toilet facility | 1. Flush toilets 2. Ventilated improved pit latrines 3. Pit latrines with slabs 4. Open pit latrines 5. No toilet facility/Bushes |
| Main floor material | 1. Earth floors 2. Dung floors 3. Cement floors |
| Main wall material | 1. Thatch walls 2. Cardboard walls 3. Bricks with cement walls 4. Bricks with mud walls 5. Poles with mud walls |
| Main roof material | 1. Thatch roofs 2. Tarpaulin roofs 3. Iron sheet roofs |
| ***Malaria prevention risk factors*** | |
| Has mosquito bed net for sleeping | 1. No 2. Yes |
| Has dwelling been sprayed against mosquitoes in last 12 months | 1. No 2. Yes |
| Has access to malaria medicine | 1. Yes 2. No |
| Child slept in Insecticide Treated Nets (ITNs) | 1. No 2. All children 3. Some children |
| ***Knowledge on the causes of malaria*** | |
| Mosquitoes bites | 1. No 2. Yes |
| Eating maize | 1. No 2. Yes |
| Eating mangoes | 1. No 2. Yes |
| Poor hygiene | 1. No 2. Yes |
| Standing water | 1. No 2. Yes |
| Not sleeping under nets | 1. Yes 2. No |
| ***Knowledge on avoiding malaria*** | |
| Sleeping under ITNs | 1. Yes 2. No |
| Taking preventative medicines | 1. Yes 2. No |
| Using mosquito repellent | 1. Yes 2. No |
| Spraying with insecticide | 1. Yes 2. No |
| Destroying breeding sites | 1. Yes 2. No |

**References**

1. Ahmed S, Reithinger R, Kaptoge S, Ngondi J. Travel is a key risk factor for malaria transmission in pre-elimination settings in Sub-Saharan Africa: A review of the literature and meta-analysis. Am J Trop Med Hyg. 2020;103(4):1380–7.

2. Björkman A, Shakely D, Ali A, Morris U, Mkali H, Abbas A, et al. From high to low malaria transmission in Zanzibar - Challenges and opportunities to achieve elimination. BMC Med. 2019;17(1):1–15.

3. Obasohan P, Walters S, Jacques R, Khatab K. A scoping review of the risk factors associated with anaemia among children under five years in sub-Saharan African countries. Int J Environ Res Public Health. 2020;17(23):1–20.

4. Boyce M, Katz R, Standley C. Risk factors for infectious diseases in urban environments of sub-Saharan Africa: A systematic review and critical appraisal of evidence. Trop Med Infect Dis. 2019;4(4):123

5. Degarege A, Fennie K, Degarege D, Chennupati S, Madhivanan P. Improving socioeconomic status may reduce the burden of malaria in sub Saharan Africa: A systematic review and meta-analysis. PLoS One. 2019;14(1):1–26.

6. Okumu F, Gyapong M, Casamitjana N, Castro M, Itoe M, Okonofua F, et al. What Africa can do to accelerate and sustain progress against malaria. PLOS Glob Public Heal. 2022;2(6):1–16.

7. Furnival-Adams J, Olanga E, Napier M, Garner P. House modifications for preventing malaria. Cochrane Database Syst Rev. 2021;2021(1)

8. Vilcins D, Sly P, Jagals P. Environmental Risk Factors Associated with Child Stunting: A Systematic Review of the Literature. Ann Glob Heal [Internet]. 2018;84(4):551–62.

9. Furnival-Adams J, Olanga EA, Napier M, Garner P. Housing interventions for preventing malaria. Cochrane Database Syst Rev. 2019;2019(8).

10. Cheng B, Htoo S, Mhote NPP, Davison C. A systematic review of factors influencing participation in two types of malaria prevention intervention in Southeast Asia. Malar J. 2021;20(1):1–9.
